# Inherent Structural Disorder and Dimerisation of Murine Norovirus NS1-2 Protein

**DOI:** 10.1371/journal.pone.0030534

**Published:** 2012-02-07

**Authors:** Estelle S. Baker, Sylvia R. Luckner, Kurt L. Krause, Paul R. Lambden, Ian N. Clarke, Vernon K. Ward

**Affiliations:** 1 Department of Microbiology and Immunology, University of Otago, Dunedin, New Zealand; 2 Department of Biochemistry, School of Medical Sciences, University of Otago, Dunedin, New Zealand; 3 Molecular Microbiology and Infection, School of Medicine, University of Southampton, Southampton, United Kingdom; Russian Academy of Sciences, Institute for Biological Instrumentation, Russian Federation

## Abstract

Human noroviruses are highly infectious viruses that cause the majority of acute, non-bacterial epidemic gastroenteritis cases worldwide. The first open reading frame of the norovirus RNA genome encodes for a polyprotein that is cleaved by the viral protease into six non-structural proteins. The first non-structural protein, NS1-2, lacks any significant sequence similarity to other viral or cellular proteins and limited information is available about the function and biophysical characteristics of this protein. Bioinformatic analyses identified an inherently disordered region (residues 1–142) in the highly divergent N-terminal region of the norovirus NS1-2 protein. Expression and purification of the NS1-2 protein of Murine norovirus confirmed these predictions by identifying several features typical of an inherently disordered protein. These were a biased amino acid composition with enrichment in the disorder promoting residues serine and proline, a lack of predicted secondary structure, a hydrophilic nature, an aberrant electrophoretic migration, an increased Stokes radius similar to that predicted for a protein from the pre-molten globule family, a high sensitivity to thermolysin proteolysis and a circular dichroism spectrum typical of an inherently disordered protein. The purification of the NS1-2 protein also identified the presence of an NS1-2 dimer in *Escherichia coli* and transfected HEK293T cells. Inherent disorder provides significant advantages including structural flexibility and the ability to bind to numerous targets allowing a single protein to have multiple functions. These advantages combined with the potential functional advantages of multimerisation suggest a multi-functional role for the NS1-2 protein.

## Introduction

Human noroviruses are highly infectious viruses that cause over 90% of all non-bacterial gastroenteritis cases worldwide [1], [2], [3]. Norovirus infections are generally self-limiting, with symptoms lasting for one to three days in healthy individuals. However, they are a significant problem for the immunocompromised and symptoms can last for up to six weeks in infants and young children [4], [5]. Every year in developing countries, noroviruses cause over one million hospitalisations and 200,000 deaths in young children [6]. In the United States alone, there are approximately 23 million cases resulting in more than 50,000 hospitalisations [7]. Noroviruses are highly transmissible; hence outbreaks are commonly in enclosed environments such as hospitals, schools and rest homes, causing widespread economic impact. The development of treatments for norovirus infection has been hindered by the inability to propagate human norovirus in cell culture; meaning limited information is available regarding the replication and biology of this virus. The *Norovirus* genus of the *Caliciviridae* contains five genogroups with multiple genotypes and subgroups [8], [9]. Genogroups I, II and IV infect humans (with genogroup II being the dominant strain worldwide) [10], while genogroups III and V infect animals. Murine norovirus (MNV; genogroup V) [10], [11] was first identified in 2003 from STAT1^−/−^/RAG2^−/−^ mice [12]. MNV is a valuable model system to study norovirus replication, as it can be easily and effectively propagated in cultured cells and a small animal model [13].

Noroviruses are non-enveloped viruses with a linear, positive-sense, single-stranded RNA genome of approximately 7.5 kb [14], [15]. The genome is modified at the 5′ end by the attachment of the viral VPg, polyadenylated at the 3′ end [12] and contains three open reading frames [15]. These encode the 187.5 kDa replicase polyprotein (*orf1*) [16], the 58.6 kDa capsid protein (*orf2*) and a small (22.1 kDa) virion-associated protein (*orf3*) [14], [17]. MNV strains also encode a fourth open reading frame (*orf4*) [11]. The non-structural *orf1* polyprotein undergoes proteolytic processing by the virus-encoded protease (NS6) to release six non-structural proteins [16]. The MNV-1 NS1-2 protein is processed further by murine caspase 3 into two fragments of 13.6 and 24.7 kDa [18]. Three of the orf1 proteins (NS5, NS6 and NS7) have been well characterised and encode for the VPg, viral protease and RNA-dependent RNA polymerase respectively. The NS3 protein encodes a putative nucleoside triphosphatase (NTPase) activity [19], while the NS4 protein of human noroviruses has been implicated in endoplasmic reticulum transport leading to Golgi disassembly and an inhibition of protein secretion [20].

The NS1-2 protein, located at the N-terminus of the replicase polyprotein, is the only protein from the replicase polyprotein that lacks significant sequence similarity to other proteins in current databases despite containing putative H box and NC motifs that suggest this protein may play a role in the regulation of cell proliferation [21]. Cellular localisation studies show that the MNV-1 NS1-2 protein co-localizes with the dsRNA within the replication complex of infected RAW26.7 cells [22] and associates with the endoplasmic reticulum and at dense punctate cytoplasmic foci when expressed in Vero cells [23]. The feline calicivirus equivalent of NS1-2 (p32) also localises to the endoplasmic reticulum [24], while the human norovirus NS1-2 protein appears to localise to the Golgi apparatus [25] and has been shown to interact with the vesicle-associated membrane protein-associated protein A (VAP-A) and affect cellular secretion [26]. It is likely that the NS1-2 protein will have multiple roles during viral replication that will be influenced by the properties of this protein.

It is becoming apparent that many eukaryotic and viral proteins are either inherently disordered (IDPs) or contain significant regions of disorder (IDRs). These regions lack a stable secondary and tertiary structure under physiological conditions [27] but are still able to carry out a wide range of functions in signaling and regulatory pathways [28]. Approximately 75% of eukaryotic signaling proteins are predicted to have long IDRs (>30 residues) while approximately 25% of all eukaryotic proteins are predicted to be fully disordered [29]. Up to 37% of eukaryotic viral proteins are predicted to contain regions of disorder and these IDRs are likely to play important roles in viral interactions with the host cell [30]. Regions of disorder are often implicated in associations with cognate ligands with this structural flexibility being important in facilitating interactions with proteins of more defined structure [29], [31].

IDRs are typified by a lack of conservation in sequence through less structural constraints upon evolution, combined with a susceptibility to protease digestion through the more relaxed structures these proteins form [32]. They also migrate aberrantly on SDS-PAGE gels due to their unusual amino acid sequence resulting in reduced SDS binding [33]. Despite the lack of a stable tertiary structure in IDPs or IDRs, these proteins still show a wide diversity in their structural properties. IDPs can exist as random coils with very little secondary structure, premolten globules (PMGs) with increased compactness and some residual secondary structure, or molten globules with increased secondary structure and compactness, (but still less than a natively folded globular protein) [32]. Computational servers can predict these disordered regions with accuracy levels higher than 69% [29]. These computational approaches are combined with several different physicochemical methods to confirm the disorder and also to distinguish between the classes of IDPs [34]. These methods include structural analyses using x-ray crystallography and NMR, circular dichroism, analysis of hydrodynamic parameters by gel filtration and dynamic light scattering, and determining susceptibility to proteolytic degradation [34].

This study uses computational approaches to show that the highly divergent N-terminal region of NS1-2 (containing the caspase 3 cleavage site [18]) of noroviruses is largely unstructured and confirms these predictions by expression, purification and characterisation of the NS1-2 protein of Murine norovirus. The complementary biophysical and biochemical studies suggest that this protein belongs to the premolten globule subfamily within the class of intrinsically disordered proteins. This study also details the presence of homodimers of recombinant NS1-2 in *Escherichia coli* and in mammalian cells.

## Results

### Secondary structure and disorder predictions of NS1-2

**Figure 1:**
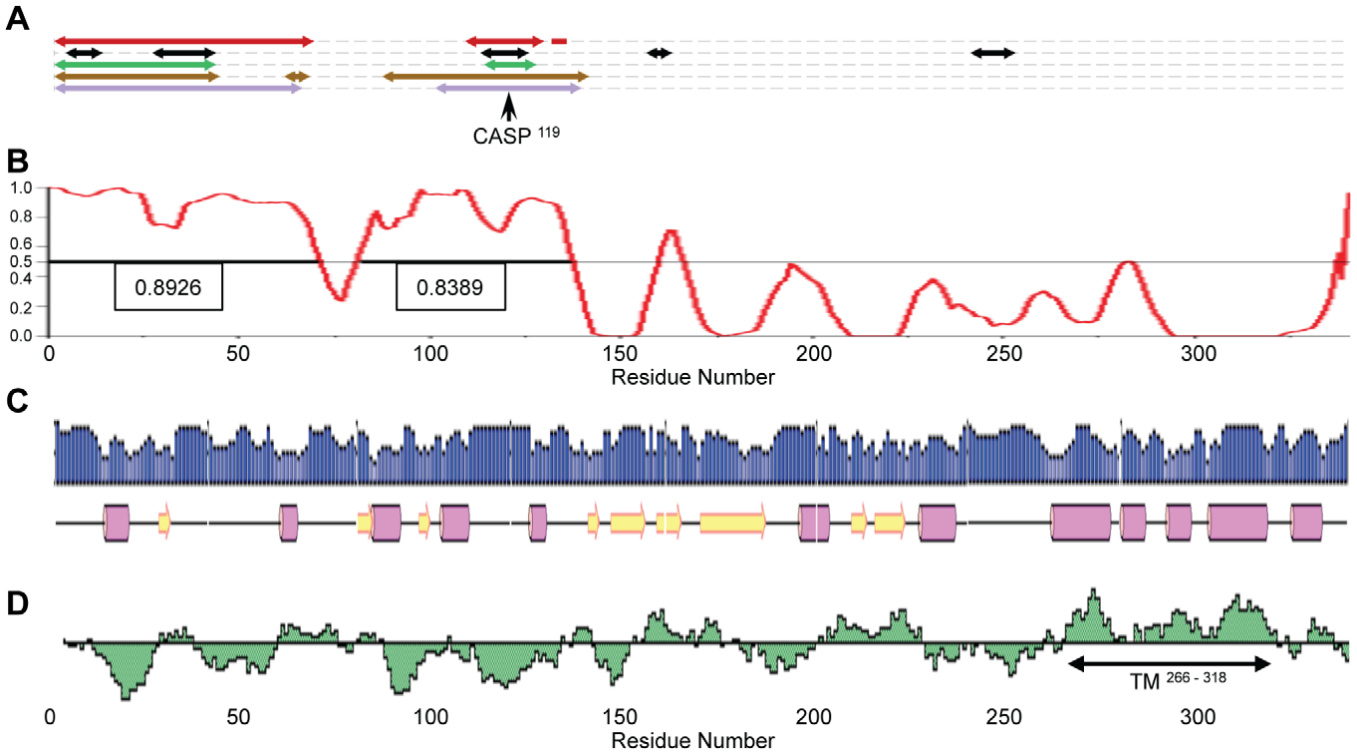
Secondary structure and disorder predictions of the MNV NS1-2 protein. (**A**) MeDor output showing five different disorder predictors with regions of disorder indicated by bi-directional arrows (IUPred – red, GlobPlot2 – black, DisEMBL – green, FoldIndex – brown, RONN – purple). CASP^119^, caspase 3 cleavage site. (**B**) PONDR® graph showing predicted disordered and ordered segments. The strength of the prediction is indicated by the PONDR® score on the y-axis. Regions above 0.5 are considered disordered [36] and are indicated by a solid black line through the central x-axis, with the corresponding average strength shown in the attached box. (**C**) PSIPRED secondary structure prediction. Pink barrels indicate helices, yellow arrows indicate strands, and the strength of the prediction is shown as the blue graph above the structural prediction (**D**) Kyte-Doolittle hydropathy plot. Hydrophobic regions are indicated above the x-axis. TM, putative transmembrane domain (residues 266–318) predicted by PSIPRED.

Bioinformatic analyses, using the Predictor of Natural Disordered Regions (PONDR®) server [35], [36], [37] and the MeDor metaserver [38], predict that most of the N-terminal 142 residues of the MNV-1 NS1-2 protein are disordered (**Fig. 1A, B**). This disordered region possesses a limited amount of secondary structure, as shown by the PSIPRED Protein Structure Prediction [39], [40] (**Fig. 1C**), and is predominantly hydrophilic (Kyte-Doolittle hydropathy plot, **Fig. 1D**). The remainder of the NS1-2 protein displays the typical features of an ordered region (increase in secondary structure and hydrophobicity), particularly in the putative transmembrane domain (residues 266–318), predicted by PSIPRED.

**Figure 2:**
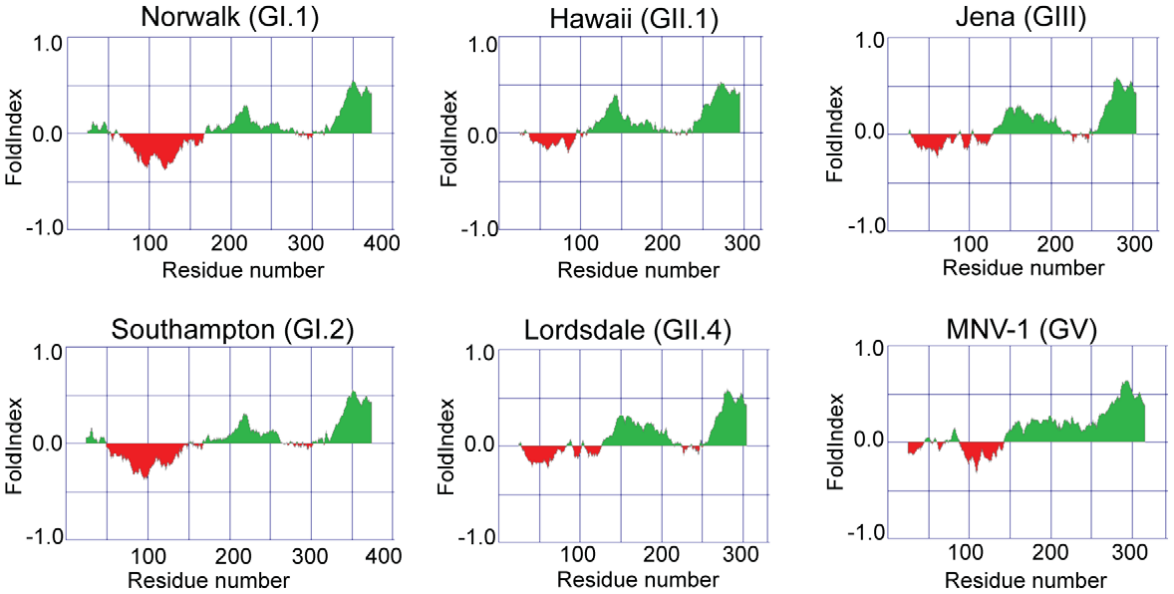
FoldIndex disorder predictions of the NS1-2 protein from norovirus genogroups. GI.1 (Norwalk), GI.2 (Southampton), GII.1 (Hawaii), GII.4 (Lordsdale), GIII (Jena) and GV (MNV-1). Ordered regions are indicated in green above 0, while disordered regions are indicated in red below 0.

**Figure 3:**
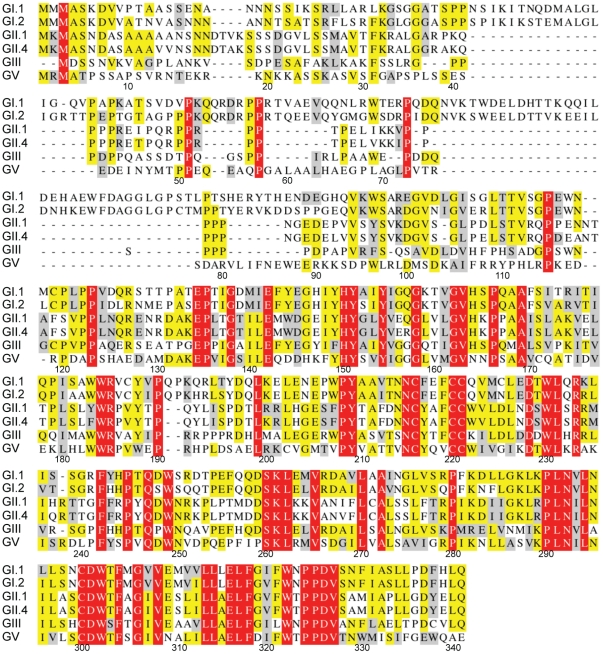
Multiple sequence alignment of the NS1-2 protein representative of different norovirus genogroups. GI.1 (Norwalk), GI.2 (Southampton), GII.1 (Hawaii), GII.4 (Lordsdale), GIII (Jena) and GV (MNV-1). Completely conserved residues are shown in white on a red background. Identical residues with >50% conservation are shaded in yellow. Similar residues with >50% conservation are shaded in grey. Residue numbers correspond to the MNV-1 NS1-2 sequence.

The prediction of disorder in the MNV-1 NS1-2 protein also occurs for the NS1-2 protein of other norovirus genogroups, including Human noroviruses, as shown by the FoldIndex [41] predictions in **Fig. 2**, confirming that this disordered region is not unique to Murine norovirus. Significant sequence divergence between related proteins is commonly observed in regions of disorder [42], [43]. Analysis of a multiple sequence alignment of the NS1-2 protein from norovirus genogroups GI.1, GI.2, GII.1, GII.4, GIII and GV showed that a marked sequence divergence does occur in the disordered region of the NS1-2 protein with the ordered C-terminal region of NS1-2 showing marked conservation (**Fig. 3**).

### Sequence properties of NS1-2

**Figure 4:**
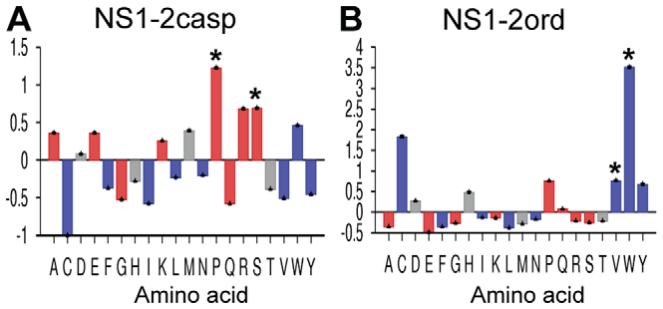
Amino acid composition analysis of the NS1-2 protein. Composition profiler analyses of NS1-2 regions showing the deviations in amino acid composition from the SWISS-PROT51 database. (**A**) N-terminal caspase cleavage product, (**B**) Middle ordered region. The relative levels of disorder promoting residues are shown as red bars, order-promoting residues are shown as blue bars and disorder neutral residues are shown as grey bars. Residues with significant enrichment (P<0.05) compared to the SWISS-PROT51 database are indicated with *.

Analysis of the amino acid sequence composition of the MNV-1 NS1-2 protein using the Composition Profiler server [44] with the SWISS-PROT51 database as a reference sample was used to determine the prevalence of order promoting or disorder promoting amino acids (**Fig. 4**). Analysis of the N-terminal caspase cleavage product, that contains the majority of the disordered region, showed enrichment in the disorder-promoting residues (proline and serine). Analysis of the middle ordered region of NS1-2 showed an increase in two order-promoting residues (valine and tryptophan).

**Figure 5:**
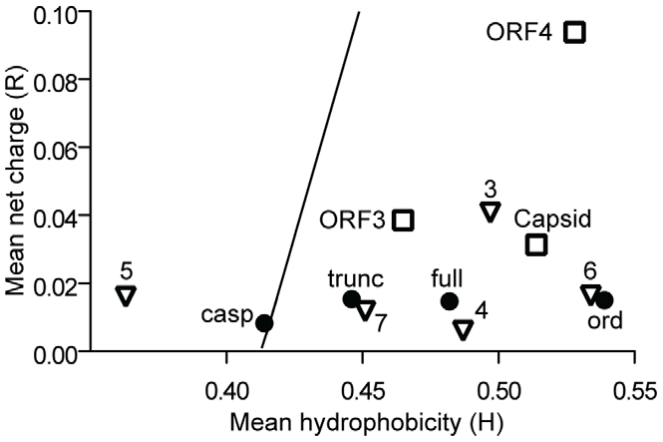
Charge-hydropathy plot of the NS1-2 protein regions and other MNV-1 proteins. The mean net charge (R) is plotted against the mean hydrophobicity (H). The boundary line is described by the equation 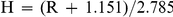. Proteins (or regions of proteins) shown to the left of the boundary line are predicted to be intrinsically disordered. Proteins to the right of the boundary line are predicted to be structured. NS1-2 regions (•); N-terminal caspase cleavage product (casp), truncated NS1-2 protein (trunc), full-length NS1-2 (full), middle ordered region (ord). The other MNV-1 non-structural proteins (∇) are numbered 2–7. Structural proteins are indicated by □.

Disordered proteins are characterised by a mean hydrophobicity/mean net charge ratio that can be shown on a charge-hydropathy plot with proteins at or left of the boundary line shown in **Fig. 5** highly likely to be disordered. [45]. The plot for the caspase cleavage product of the NS1-2 protein (NS1-2casp) lies just to the left of the boundary line indicating disorder. Closer analysis of the mean hydrophobicity of the NS1-2casp protein, shows that it is 0.004 units from the boundary (H_boundary_ – H_casp_), consistent with the values expected for an IDP from the pre-molten globule (PMG) family (0.037±0.033) [32]. The other NS1-2 regions are predicted to be ordered, as are all of the other ORF1 proteins with the exception of NS5 (VPg). FoldIndex [41] analysis of the VPg region also predicts this to be significantly disordered, as has been detailed for the VPg protein of other viruses [46]. The three structural proteins (ORF2 (capsid), 3, 4) all lie to the right of the boundary line indicating order.

### Expression and purification of NS1-2

**Figure 6:**
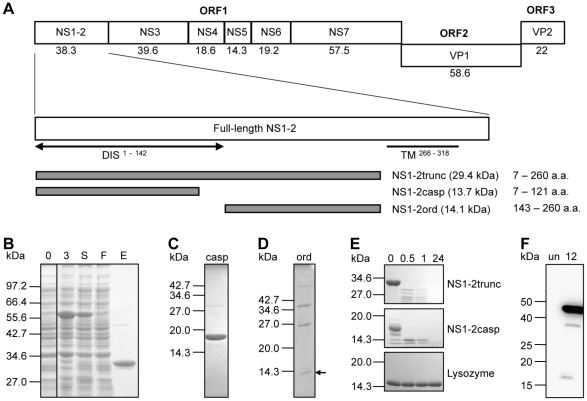
Protein design and expression of the MNV-1 NS1-2 protein in *Escherichia coli*. (**A**) Schematic diagram of the MNV genome and the NS1-2 protein. TM, predicted transmembrane domain. DIS, disordered region. The expressed regions (truncated, caspase cleavage product and middle ordered region) are indicated by amino acid number (a.a.) and the molecular masses (in kDa) are indicated below or beside each protein. (**B**) SDS-PAGE analysis of the expression and purification of the NS1-2trunc region. The CBD-Intein-NS1-2trunc fusion protein is visible at three hours post-induction and in the soluble fraction. The NS1-2trunc protein is shown in the elution fraction collected after cleavage of the intein. Marker, NEB Broad Range. 0, Pre-induction. 3, three hours post-induction. S, soluble. F, flow through from the chitin bead column. E, elution. The vertical line indicates that two sections of the same gel have been combined in this figure. (**C**) SDS-PAGE analysis of the eluted fraction collected from the chitin bead columns for NS1-2casp. (**D**) SDS-PAGE analysis of the fraction collected after stripping the chitin column of NS1-2ord. (**E**) SDS PAGE analysis showing thermolysin digestion of each of the NS1-2 protein fragments. Lysozyme was used as globular protein control, showing resistance to proteolysis even at 24 hours. 0, sample collected before adding thermolysin. 0.5, 30 minutes digest. 1, one-hour digest. 24, 24-hour digest. (**F**) Western blot analysis of MNV-infected RAW264.7 cells using a 1 in 2500 dilution of the rabbit polyclonal NS1-2 antibody stock. The antibody detects the NS1-2 full-length protein (actual size of 38.3 kDa, observed at ∼44 kDa) and caspase 3 cleavage products of 24.7 kDa (observed at ∼30 kDa) and 13.6 kDa (observed at ∼18 kDa). Marker, Invitrogen BenchMark™ Pre-stained Protein Ladder. 12, RAW264.7 cells harvested at 12 hours post-infection with MNV-1. Un, RAW264.7 cells only (negative control).

To experimentally confirm the bioinformatic predictions of disorder, we have expressed, purified and characterised the truncated MNV-1 NS1-2 fragment (minus the transmembrane domain), the disordered N-terminal caspase fragment and the ordered region of the truncated construct in the NEB IMPACT™-TWIN system (**Fig. 6**). The truncated fragment (NS1-2trunc) and the caspase fragment (NS1-2casp) were purified successfully (**Fig. 6B,C**), however the ordered fragment (NS1-2ord) did not elute from the chitin column and could only be visualised by column stripping with 1% SDS (**Fig. 6D**). The identity of the purified recombinant proteins was confirmed by mass spectrometry analysis.

The recombinant NS1-2 proteins containing a significant region of disorder (NS1-2trunc and NS1-2casp) migrated slower than the associated theoretical molecular mass on SDS-PAGE (**Fig. 6B–C** and **Table 1**). This is also observed for the full-length NS1-2 protein in MNV-infected RAW264.7 cells, when analysed by western blot analysis using polyclonal rabbit serum raised against the purified NS1-2trunc protein (**Fig. 6F**). As the percentage of disorder increased, the inhibition of migration also increased as shown by the increase in the ratio between the theoretical and apparent molecular masses (**Table 1**). The fraction containing no disordered residues (NS1-2ord) showed normal migration (**Fig. 6D**). The purified NS1-2trunc and NS1-2casp proteins were sensitive to digestion by thermolysin compared to the globular lysozyme control (**Fig. 6E** **)**, with obvious degradation visible by SDS-PAGE after only thirty minutes.

**Table 1:** Migration of the NS1-2 regions on SDS-PAGE.

	MM theo^1^ (kDa)	MM app^2^ (kDa)	Ratio
NS1-2full	38.3	44	1.17
NS1-2trunc	29.4	34	1.19
NS1-2casp	13.7	18	1.31
NS1-2ord	14.1	14	none

1 The theoretical molecular mass (MM theo) is based on the amino acid sequence of the expressed protein.

2 The apparent molecular mass (MM app) was determined by SDS-PAGE analysis.

### NS1-2 forms homodimers

**Figure 7:**
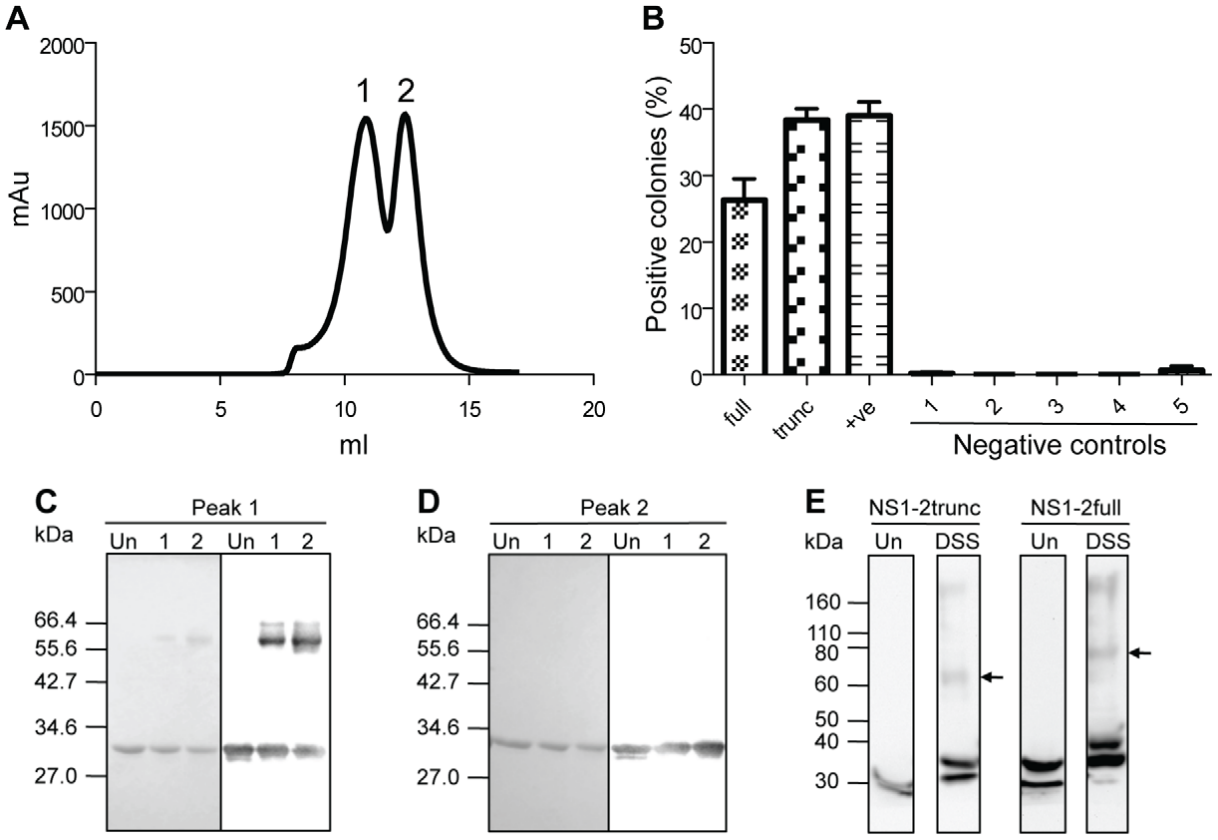
Dimerisation of the NS1-2 protein. (**A**) Chromatogram showing the two peaks (1 and 2) obtained during purification of the NS1-2trunc protein through a Superose12 column. (**B**) Bacterial two-hybrid analyses show a positive interaction between both full-length NS1-2 and truncated NS1-2 clones. full, pBT-NS1-2full + pTRG-NS1-2full. trunc, pBT-NS1-2trunc + pTRG-NS1-2trunc. +ve, positive control, pBT-LGF2 + pTRG-Gal11^P^. 1, negative control for medium quality (pTRG- Gal11^P^ + pBT). 2, pBT-NS1-2full + pTRG. 3, pTRG-NS1-2full + pBT. 4, pBT-NS1-2trunc + pTRG. 5, pTRG-NS1-2trunc + pBT. (**C**) 10% SDS-PAGE (left) and western blot (right) of GA cross-linking of NS1-2trunc from peak 1 of the size exclusion column. (**D**) 10% SDS-PAGE (left) and western blot (right) of GA cross-linking of NS1-2trunc from peak 2 of the size exclusion column. The NS1-2 monoclonal antibody was used for western blot analysis in Fig. C and D. (**E**) Western blot analysis of HEK293T cells harvested 24 hours post-transfection with the NS1-2 protein constructs. Arrows indicate the NS1-2 dimer band for each construct. The NS1-2 polyclonal antibody was used at a 1 in 5000 dilution for the detection by western blot. Legend for Fig. C, D and E: Markers, NEB Broad Range (SDS-PAGE gels), Invitrogen Novex® Sharp Protein Standard (western blots). Un, untreated protein. 1, cross-linked with 0.005% GA. 2, cross-linked with 0.01% GA. DSS, cross-linked with 5 mM DSS.

As part of the purification required for biophysical analysis, the MNV-1 NS1-2trunc protein was purified through a Superose12 size exclusion column. This resulted in the appearance of two distinct peaks suggesting multimerisation of the protein (**Fig. 7A**). The addition of DTT to the column buffer (1 mM) and protein sample (2 mM) had no effect on multimerisation of the NS1-2trunc protein, indicating that disulphide bonds are not involved in this multimerisation. Protein samples collected from each of these peaks were cross-linked using glutaraldehyde (GA) and analysed by SDS-PAGE and western blot (**Fig. 7C,D**). The NS1-2trunc protein is visible at ∼34 kDa in samples from both size exclusion column peaks, as well as a band with an observed molecular mass of ∼66 kDa from the higher molecular mass peak. This ∼66 kDa band corresponds to a dimer of the NS1-2trunc protein. The higher observed molecular mass of the dimer band at ∼66 kDa (compared to the actual mass of 58.8 kDa) once again reflects the slower migration of the NS1-2 protein. Intact mass analysis of samples collected from the dimer and monomer peaks by mass spectrometry on a MALDI-TOF/TOF identified the presence of a major peak at ∼58.8 kDa for the dimer sample, which was absent from the monomer sample (29.4 kDa).

The BacterioMatch® II bacterial two-hybrid system (Stratagene, Agilent Technologies, La Jolla, CA) provided further evidence that the MNV-1 NS1-2 protein can form a dimer. Transcriptional activation was observed between both full-length NS1-2 constructs (26% positive) and NS1-2trunc constructs (38% positive) (**Fig. 7B**). This positive interaction was detected on selective medium containing 3 mM 3-AT but not detectable on medium containing 5 mM 3-AT, indicating that the interaction may have been too weak to overcome the high competitive inhibition of 5 mM 3-AT.

To investigate if the NS1-2 multimerisation also occurred in a mammalian cell line, the full-length and truncated NS1-2 constructs were expressed in HEK293T cells under the control of a CMV promoter. At 24 hours post-transfection (hpt) the cells were harvested and the lysate cross-linked with disuccinimidyl suberate (DSS). DSS is a membrane permeable cross-linker. Western blot analysis identified a higher molecular mass band corresponding to the size of the dimer for each construct (**Fig. 7E**). The higher molecular mass band (∼200 kDa) present in both cross-linked samples has yet to be characterised.

### The NS1-2 protein is an elongated protein

Calibration of the Superose12 size exclusion column indicated that the NS1-2trunc monomer fraction was migrating as an approximately 70 kDa protein and the dimer fraction at approximately 190 kDa. Both of these values are substantially larger than the theoretical molecular masses (from amino acid sequence) of 29.4 kDa and 58.8 kDa respectively. However, these values are obtained on the assumption that the NS1-2trunc protein is a globular protein, which is unlikely based on the bioinformatic predictions of disorder. The same elution profiles from the size exclusion column were observed independent of protein loading, NaCl concentration, pH, or elution buffer (Tris or Citrate-phosphate).

To resolve this discrepancy, the Svedberg equation [47] was used to obtain an experimental measure of the molecular mass of a protein based on an observed Stokes radius (R_s_) and sedimentation coefficient (S). The Stokes radii (R_s_) of the NS1-2trunc monomer and dimer fractions were calculated (using the Porath Solution, described in [48]) to be 3.51 nm (35.1 Å) and 5.17 nm (51.7 Å) respectively. Very similar R_s_ values (3.52 nm and 5.15 nm respectively) were calculated using the alternative approach of Laurant and Killander [48].

Dynamic light scattering (DLS) was used to confirm these R_s_ values and ensure monodispersity of each protein sample. DLS data predicted average R_s_ values of 35.4 Å for NS1-2trunc monomer, 55.3 Å for the NS1-2trunc dimer and 24.3 Å for the NS1-2casp protein, comparable to the R_s_ values predicted from the size exclusion column (**Table 2**).

**Table 2:** Stokes radii observations and predictions for the expressed regions of NS1-2.

	Observed R_S_ (Å)		Predicted R_S_ (Å)^3^			
	SEC^1^	DLS^2^	NF	MG	PMG	UN
NS1-2trunc monomer	35.1	35.4±5.9	24.6	27.5	34.8	47.8
NS1-2trunc dimer	51.7	55.3±7.3	31.5	34.7	45.7	68.5
NS1-2casp	23.3	24.3±0.6	18.7	21.3	25.8	32.0

1 The observed Stokes radii (R_S_) from the Superose12 size exclusion column (SEC) were determined from the standard curve (r^2^ = 0.9820) obtained after calibration of the column with the standards from the Sigma MW-GF-200 kit.

2 The R_S_ value from dynamic light scattering (DLS) is the average value ± SD obtained from five (NS1-2trunc monomer) and three (NS1-2trunc dimer and NS1-2casp) independent measurements.

3 The predicted R_S_ values were calculated for each possible protein conformation from the equations described in the *Hydrodynamic characterization* methods section. NF, natively folded. MG, molten globule. PMG, premolten globule. UN, unfolded in urea.

The sedimentation coefficients (S) of the NS1-2trunc monomer and dimer were determined by the separation of the NS1-2trunc protein and standards through a sucrose gradient. The linear equation of 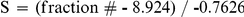 (r^2^ = 0.9997) was obtained from the standards and used to calculate the approximate S values of 2.0 (monomer) and 2.9 (dimer) for the NS1-2trunc protein. The molecular masses of the NS1-2trunc fractions were calculated (using the simplified Svedberg equation [47]) as 29.5 kDa (monomer) and 63.0 kDa (dimer), consistent with the theoretical masses of 29.4 kDa and 58.8 kDa respectively.

The shapes of the NS1-2trunc monomer and dimer were determined by calculating the ratio between the maximum possible sedimentation coefficient (S_max_) and the observed sedimentation coefficient (S). The S_max_ values determined as described in [47] resulted in S_max_/S ratios of 1.72 for the monomer and 1.88 for the dimer, indicating that NS1-2trunc is a moderately elongated protein.

The predicted R_s_ values for each possible conformation of NS1-2trunc (monomer and dimer) and NS1-2casp were determined, as described in [49] and compared to the observed R_s_ values (obtained from the size exclusion column and dynamic light scattering). Each NS1-2 protein construct was shown to have an observed R_s_ value similar to the expected value of a pre-molten globule (PMG) intrinsically disordered protein (**Table 2**).

### CD spectra of the NS1-2 protein are typical of a PMG protein

**Figure 8:**
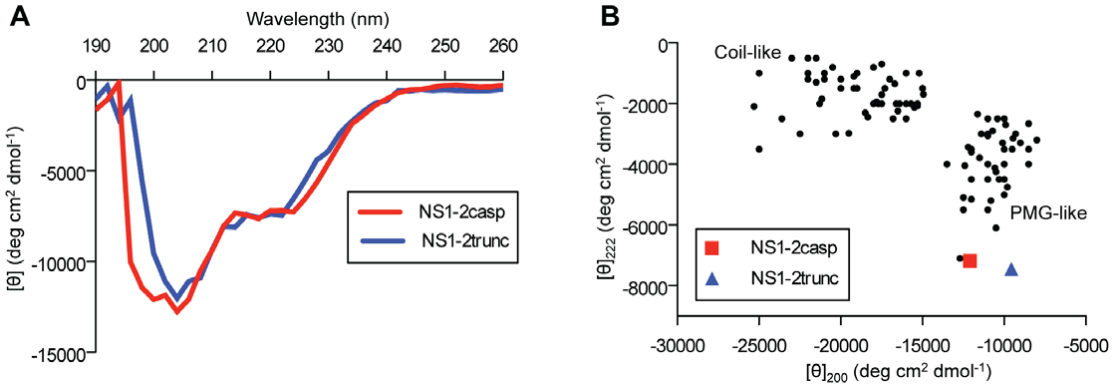
Analysis of the NS1-2 protein by far-UV circular dichroism. (**A**) Far-UV CD spectra of NS1-2trunc and NS1-2casp in 20 mM citrate-phosphate pH 6.1, 150 mM NaCl. The CD spectra are the average of five independent acquisitions. (**B**) Double wavelength plot, [θ]_222_ versus [θ]_200_, of a set of ‘natively unfolded’ proteins (from [32]) and the NS1-2trunc and NS1-2casp proteins.

The far-UV circular dichroism (CD) spectra of the NS1-2trunc and NS1-2casp purified proteins are typical of a PMG protein, as seen from the large negative ellipticity at ∼200 nm and low ellipticity at 190 nm (**Fig. 8A**). Interestingly the ellipticity remains lower at ∼222 nm than compared to many other unfolded proteins [32], indicating residual secondary structure. The ellipticity values at 200 and 222 nm for the NS1-2 proteins were also plotted alongside other disordered proteins belonging to the random coil and PMG families (**Fig. 8B**). This provided further evidence that the NS1-2 protein is a PMG type of disordered protein. Analysis of the CD data using the DichroWeb CD server deconvolution methods [50], [51], [52], [53] indicated disorder proportions of 40–56%.

## Discussion

Bioinformatic analyses of the MNV NS1-2 protein identified a region at the N-terminus of this protein that had the typical features of an inherently disordered region (IDR), including a limited amount of secondary structure and an overall hydrophilic nature, both typical features of IDPs [29]. Six individual bioinformatic servers were used to identify the disordered region showing a good consensus across all predictors. Inherently disordered proteins have been shown to have a biased amino acid composition, with a depletion in order-promoting residues such as W, C, F, Y, I, V, or L and an enrichment in disorder-promoting residues (A, R, Q, S, P, or E) [36], [54]. The comparison of the overall hydrophobicity and net charge of a protein region is another useful approach for predicting for disorder [45]. The N-terminal region of NS1-2 represented by the caspase 3 product (NS1-2casp), has a significant enrichment in the disorder-promoting residues proline and serine and has a mean hydrophobicity/mean net charge ratio that is typical of an IDP from the PMG family [45]. The middle ordered region, NS1-2ord, has a significantly higher mean hydrophobicity placing it well above the boundary line. Previous analyses using this boundary line equation [49] have indicated that there is a very low positive error rate (globular proteins wrongly assigned as disordered), and an ∼5% negative error rate (disordered wrongly assigned as ordered) [42]. Within this IDR of the NS1-2 protein, there is a short region that has low sequence complexity. Small ordered sections are often observed in extended regions of structural disorder [36], [42] and may be Molecular Recognition Features (MoRFs) that gain a stable structure induced by binding to a partner or ligand [55].

Bioinformatic analyses of the NS1-2 protein of other norovirus genogroups, has shown that these other NS1-2 proteins also possess N-terminal IDRs. The marked sequence divergence between the IDRs of noroviruses is consistent with the sequence variability observed in disordered regions [42], [43]. There are four proposed reasons for this increased variability [43]: *(i)* a difference in amino acid composition (less aromatic and more charged amino acids in disordered regions), *(ii)* unconstrained evolution due to the region having no function (however, many known disordered proteins do have functions), *(iii)* no fixed structure gives a function (e.g. flexible linkers) and *(iv)* positive selection for variability [43]. The function of the MNV NS1-2 protein is currently unknown; hence it is difficult to determine why this protein contains an IDR. However, it seems likely that like many viral proteins, NS1-2 may perform multiple roles during viral replication and a disordered region would enhance the flexibility of this protein.

In agreement with these IDR-typical sequence properties, the NS1-2 protein showed several biophysical features typical of an IDR, notably an aberrant electrophoretic migration [33], increased protease sensitivity [56], increased hydrodynamic radius (Stokes radius) [49] and far-UV spectra typical of an IDR from the PMG family [32]. The abnormal electrophoretic migration of the NS1-2 was more pronounced as the percentage of disorder increased, with the NS1-2casp protein migrating approximately 1.31 times larger than expected. The aberrant migration of the NS1-2 protein was also observed in MNV-1-infected RAW264.7 cells, providing evidence that the NS1-2 protein is also inherently disordered in vivo. Aberrant electrophoretic migration can be due to post-translational modifications (such as phosphorylation and glycosylation), binding of less SDS (due to a high hydrophilicity and/or a high number of negatively charged amino acids) [33], or a high proline content. Proline is the strongest disorder promoting residue [57], due to its helix breaking nature [55], [58], [59]. The aberrant migration of the NS1-2 protein is predicted to be due to both the hydrophilic nature of the disordered region and the high proline content across the whole protein (8.5%). IDPs have an increased protease sensitivity due to the extended nature of the proteins, the lack of a packed core [56] and increased solvent accessibility compared to globular proteins [42]. The protease sensitivity of the NS1-2 protein is shown by a high sensitivity to digestion with thermolysin, a protease with broad substrate specificity. The previously documented caspase 3 cleavage of the NS1-2 [18] also occurs in a region with a strongly disordered nature.

The increased Stokes radius (R_s_) of the NS1-2 protein determined from gel filtration and dynamic light scattering analyses are consistent with the R_s_ values predicted for an IDP from the PMG family [49]. The linear equations that were used to calculate these predicted values relate molecular mass to R_s_ and were generated from the analysis of over a hundred well-characterised proteins [32]. Furthermore, determination of the ratio between the maximum possible sedimentation coefficient (S_max_) and observed sedimentation coefficient (S) of the NS1-2trunc protein showed that it is a moderately elongated protein [47], a typical feature of IDPs [49]. Calibration of the Superose12 size exclusion column indicated that the two peaks observed for the NS1-2trunc protein corresponded to two proteins of approximately 70 kDa and 190 kDa, implying that the NS1-2trunc protein was migrating as a dimer and a higher oligomer (possibly hexamer). However, we were able to show, using the Svedberg equation that the NS1-2trunc protein was in fact migrating as a monomer and dimer. The Svedberg equation combines independent information obtained from the sedimentation coefficient and Stokes radii to increase the accuracy of the predicted molecular mass measurement, regardless of the protein conformation. At no point in the equations is the theoretical molecular mass of the protein entered, hence avoiding any bias towards this value. This equation is very useful to determine the multimeric state(s) of a protein in solution, as it has been used here to show that the recombinant NS1-2trunc protein exists as both a monomer and dimer in solution. Intact mass analysis by mass spectrometry of samples collected from the monomer and dimer peaks provided further confirmation of the monomeric and dimeric molecular masses. The far-UV spectra further confirm that the NS1-2 protein belongs to the PMG family of IDPs, as the parameters indicate that they possess some residual secondary structure, typical of the PMG conformation [32].

During the biophysical analysis of the NS1-2 protein, it was discovered that the truncated and full-length forms of the protein are able to form dimers. The first indication of this multimerisation was during purification of the NS1-2trunc protein through a size exclusion column where two distinct peaks were observed. This multimerisation was confirmed by chemical crosslinking, intact mass spectrometry and a bacterial two-hybrid assay. Purification of the NS1-2casp protein through the size exclusion column resulted in a single peak that was shown to be a monomer. This indicates that the multimerisation domain is not localised solely to the N-terminal disordered region of the protein. Some proteins, including the norovirus RNA-dependent RNA polymerase [60] must be in a multimeric form to be active, while other proteins have differing functions depending on their oligomeric state.

Inherent disorder provides several advantages that have been well described in recent reviews [29], [61]. IDRs are involved in numerous protein-protein, protein-nucleic acid and protein-ligand interactions that occur in vital biological processes, particularly in signaling and regulatory pathways [28], [29], [62]. Inherent disorder allows a specific protein region to have increased flexibility and multiple functions [42], such as the genome-linked viral protein, VPg [46]. IDRs are able to bind to numerous targets [29], [63], [64] with both a high specificity and low affinity [27], [59], [65], [66], [67]. Generally when this binding occurs, a short region of the IDR (commonly less than 30 residues) develops an ordered secondary structure [61], [68]. Although IDRs have low sequence conservation, this short binding region often shows much higher conservation [69]. The flexible nature of disordered proteins allows for an increased speed of interactions with binding partners. The high protease sensitivity of elongated disordered regions also enables more efficient regulation of protein levels, which is particularly important in regulatory pathways [58]. Viral proteins, especially from RNA viruses, are significantly enriched in IDRs [70], and it has been proposed that these IDRs are able to buffer the detrimental effects of the high mutation rate (10^−5^–10^−3^ [71]) in RNA viruses [70]. The ability of IDRs to have multiple functions also has the added advantage of enabling the virus to retain a compact genome while still providing all the necessary functions for successful replication [42].

Inherent disorder and the potential functional advantages of multimerisation suggest that the NS1-2 protein of noroviruses may have several different functions. It has previously been indicated that the MNV-1 NS1-2 protein plays a role in membrane recruitment during the formation of the MNV replication complex [22], [23] while the NS1-2 protein of Norwalk virus has been linked to the control of protein secretion [20]. Another multifunctional non-structural viral protein with inherently disordered regions is the NS5A protein of Hepatitis C virus. The Hepatitis C virus NS5A protein has proline-rich hydrophilic and inherently disordered regions and binds to a number of cellular proteins [72]. Recent clinical trials using a high affinity anti-viral targeted towards the NS5A protein have shown a clear reduction in virus numbers in patients chronically infected with Hepatitis C virus [73]. The disordered nature and potential multi-functional nature of the norovirus NS1-2 protein indicate that this protein could make a good drug target against norovirus infections, analogous to the NS5A protein of Hepatitis C virus.

## Materials and Methods

### Predictions of secondary structure and disorder

The predictions of disorder in the NS1-2 protein were obtained using the Predictor of Natural Disordered Regions (PONDR®) server [35], [36], [37] (http://www.pondr.com/) and the Metaserver of Disorder (MeDor) [38] (http://www.vazymolo.org/MeDor/index.html). The PONDR® output is shown graphically as a plot indicating the strength of the prediction at each region. The MeDor metaserver generates a graphical output showing secondary structure and disorder predictions from servers freely available on the web, hence showing the predictions from multiple different analyses. **Fig. 1B** shows the disorder predictions from IUPred [74], GlobPlot2 [75], DisEMBL [76], FoldIndex [41] and RONN [77].

The PSIPRED Protein Structure Prediction Server [39], [40] (http://bioinf.cs.ucl.ac.uk/psipred/) was used as a predictor of secondary structure (PSIPRED v3.0 [39]) and transmembrane topology prediction (MEMSAT3 and MEMSAT-SVM [78], [79]). Kyte-Doolittle hydropathy plots were generated on the Protean application from the Lasergene® suite of the DNASTAR sequence analysis software (DNASTAR Inc., Madison, WI, USA). The MegAlign application from the Lasergene® suite was used to align the NS1-2 protein sequences from MNV-1 (GV, DQ285629), Norwalk (GI.1, M87661), Southampton (GI.2, L07418), Hawaii (GII.1, U07611), Lordsdale (GII.4, X86557) and Jena (GIII, EU360814) using the ClustalW method. The alignment (in PAUP [Nexus] format) was presented using the Mobyle@Pasteur v1.0 portal (http://mobyle.pasteur.fr/cgi-bin/portal.py?#welcome) developed jointly by the Institut Pasteur “Projets et Développements en Bioinformatique” Team and the Ressource Parisienne en Bioinformatique Structurale.

### Analysis of amino acid composition and charge-hydropathy plots

The Composition Profiler server [44] (http://www.cprofiler.org/) was used to identify any deviations in amino acid composition compared to the SWISS-PROT51 database. Charge-hydropathy (CH) plots were generated as described previously [32], [42]. The ProtParam [80] program at the EXPASY server (http:/web.expasy.org/protparam) was used to determine the number of positively and negatively charged amino acids at pH 7 for the proteins shown on the CH plot. The mean net charge (R) was calculated by determining the value of the absolute difference between the positively and negatively charged residues and dividing this by the total number of residues. The Protscale program [80] at the EXPASY server (http://web.expasy.org/protscale) was used to calculate the individual hydrophobicities using the options ‘Hphob/Kyte & Doolittle’, window size = 5, and normalizing the scale from 0 to 1. Summing these individual hydrophobicities and dividing by the total number of residues minus 4 calculated the mean hydrophobicity (H). Plotting H versus R generated the CH plot. The boundary line corresponds to the equation 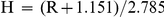, which defines the boundary between disordered proteins (left side) and ordered proteins (right side) [32].

### Cloning, expression and purification of NS1-2 in *E. coli*

MNV NS1-2 clones were generated as N-terminal fusion constructs in the pTWIN1 vector of the IMPACT™-TWIN expression system (New England Biolabs Inc., Beverly, MA, USA). The first three residues of NS1-2 (Met, Arg, Met) were excluded from the NS1-2trunc and NS1-2casp clones due to the inhibitory affect of the two methionine residues during the intein cleavage. The 5′ primers were designed to include an *NcoI* site (GGTGGTCCATGGTGGCAACGCCATCTTCTGC – NS1-2trunc and NS1-2casp, GGTGGTCCATGGTACAGGATGATCACAAGTTT – NS1-2ord). The 3′ primers included a stop codon and a *PstI* site (GGTGGTCTGCAGTTAGGATGGAATGAAGGGCTC – NS1-2trunc and NS1-2ord, GGTGGTCTGCAGTTAGTCAGGCCTATCCTCCTTAG – NS1-2casp). The PCR products were generated with Expand Polymerase (Roche Diagnostics, New Zealand Ltd, Auckland, NZ) from an MNV-1 template, purified (AxyPrep™ PCR Cleanup Kit, Axygen Biosciences, Union City, CA, USA), ligated into pGEM®-T Easy (Promega Corporation, Madison, WI, USA) for sequence confirmation (Allan Wilson Genome Sequencing Centre, Albany, NZ) and then subcloned into pTWIN1. Cloning into the *NcoI* site of pTWIN1 introduced three extra amino acids (Gly, Arg, Ala) at the N-terminus of each of the recombinant proteins. Protein expression and purification was as per manufacturer's instructions. Briefly, expression was achieved in C41(DE3) *E. coli* by growth in Luria broth medium (containing 50 µg/ml ampicillin) at 37°C to an OD_600_ reading of 0.6, before inducing with 0.5 mM IPTG and continuing expression at 25°C for three hours. Cell pellets (collected from 500 ml of culture) were resuspended in 25 ml of 20 mM HEPES pH 8.5, 1 M NaCl, 1 mM EDTA containing 100 µg DNaseI and 0.1% Tween-20 and lysed by French press. The soluble fraction was purified through a chitin bead gravity chromatography column with fusion protein cleavage occurring overnight at room temperature in 20 mM HEPES pH 6.0, 1 M NaCl, 1 mM EDTA, before elution the following day.

### Concentrating proteins and visualisation by SDS-PAGE and western blot

The NS1-2 proteins were concentrated at 4°C using Amicon® Ultra and Microcon® centrifugal filter devices (Millipore) with molecular weight cut-off values of 10,000 Da. All protein fractions were stored at either −80°C for long-term storage or at 4°C for a maximum of two days. Protein concentrations were determined on a NanoDrop 1000 Spectrophotometer (Thermo Scientific) using the theoretical extinction coefficients at 280 nm obtained from the ProtParam program at the EXPASY server of 56,000 (NS1-2trunc), 13,980 (NS1-2casp) and 42,000 (NS1-2ord).

Protein visualisation was achieved by separation on SDS-PAGE gels and either staining with Coomassie Blue, or transferring to a PVDF membrane for subsequent detection by western blotting. Primary antibodies were either the NS1-2 polyclonal antibody generated in rabbits (at a 1 in 2500 or 5000 dilution in 1% casein alanate in PBS) or the NS1-2 monoclonal antibody, provided by Professor Ian Clarke, Molecular Microbiology, University of Southampton (at a 1 in 20 dilution). Secondary antibodies were either anti-rabbit-HRP or anti-mouse-HRP (Sigma). Both secondary antibodies were used at a 1 in 5000 dilution in 1% casein alanate. Gel and western membrane images were captured on a BioRad ChemiDoc gel documentation system (BioRad, Hercules, CA, USA).

### Generation of polyclonal antibodies

Rabbits were vaccinated with 200 µg of purified NS1-2trunc protein in Freund's complete adjuvant (Sigma-Aldrich Pty Ltd, Castle Hill, NSW, Australia) then boosted twice at three-week intervals with 200 µg of protein in Freund's incomplete adjuvant. The antibody was screened and dilutions optimised using MNV-infected RAW264.7 cells (TIB-71™, American Type Culture Collection (ATCC), Manassas, VA, USA).

### Digestion of NS1-2 by thermolysin

A stock solution of thermolysin was prepared at 0.4 mg/ml (Sigma, 50–100 units/mg) in 10 mM Tris pH 8, 300 mM NaCl, and stored at −20°C. NS1-2 protein samples (at 8–13 mg/ml in 20–50 mM citrate phosphate pH 6.1, 150 mM NaCl) were diluted to 1 mg/ml in 10 mM Tris pH 8, 300 mM NaCl and digested with thermolysin for 24 hours at 26°C. Lysozyme was used as a globular protein control. Thermolysin: NS1-2 protein ratios were 1∶100 (w/w). Samples were collected at time points of 0, 30 minutes, 60 minutes and 24 hours and the extent of proteolysis was visualised on 12.5% SDS-PAGE gels.

### Size exclusion analysis

The fractions collected from the chitin column were concentrated to 10–20 mg/ml (in 500 µl total volume) and subjected to size exclusion chromatography at 4°C, on a 24 ml Superose12 column. This column was equilibrated with 20–50 mM citrate phosphate pH 6.1, 150 mM NaCl. A flow rate of 0.5 ml/min was used to elute the purified protein in 300 µl fractions. The column was calibrated using blue dextran, cytochrome c, β-amylase, carbonic anhydrase, alcohol dehydrogenase and albumin, as per the protocol from the Sigma MW-GF-200 kit.

### Mass spectrometry analysis

The identity of protein bands on SDS-PAGE gels was determined by MALDI tandem time-of-flight mass spectrometry at the Centre for Protein Research, University of Otago, New Zealand. Protein bands were excised from the gel, digested with trypsin according to the method of Shevchenko et al. [81] and eluted peptides dried using a centrifugal concentrator. Peptides were resuspended in 30% (v/v) aqueous acetonitrile containing 1% (v/v) trifluoroacetic acid and 1 µl mixed with 2 µl of matrix (10 mg/ml of α-cyano-4-hydroxycinnamic acid dissolved in 65% (v/v) aqueous acetonitrile containing 1% (v/v) trifluoroacetic acid and 10 mM ammonium dihydrogen phosphate). An aliquot (0.8 µl) of this was spotted onto a MALDI Opti-TOF 384 well sample plate (Applied Biosystems™ by Life Technologies, Carlsbad, CA, USA) and air-dried. Samples were analysed on a 4800 MALDI-TOF/TOF analyser (Applied Biosystems™) and the MS spectra were acquired in linear positive-ion mode with 1200 laser pulses per sample spot. Proteins were identified by using the MS/MS data to search against the UniProt/Swiss-Prot amino acid sequence database in the Mascot search engine (http://www.matrixscience.com). The searches were set up for full tryptic peptides allowing for three missed cleavages, carboxyamidomethyl cysteine and oxidised methionine as variable modifications and mass tolerance levels of 75 ppm (peptide mass from MS data) and 0.4 Da (fragment ions from MS/MS data).

For the intact mass analysis of the NS1-2trunc monomer and dimer proteins, the protein samples were collected from the centre of each peak on the size exclusion column and concentrated to ∼5 mg/ml (150 pmol/µl) in 50 mM citrate phosphate pH 6.1, 150 mM NaCl. Each sample was diluted either 1∶20 (dimer) or 1∶100 (monomer) in 30% (v/v) aqueous acetonitrile containing 1% (v/v) trifluoroacetic acid and 1 µl mixed with 1 µl of matrix (10 mg/ml of α-cyano-4-hydroxycinnamic acid dissolved in 65% (v/v) aqueous acetonitrile containing 1% (v/v) trifluoroacetic acid). An aliquot (0.8 µl) of this was spotted onto a MALDI Opti-TOF 384 well sample plate and air-dried. Samples were analysed on the MALDI-TOF/TOF analyser as described above. The mass range of the MALDI-TOF/TOF had been calibrated on a 5-peptide/protein-calibration mix (1000 to 25,000) and on the BSA 1+ and 2+ ions (20,000 to 100,000).

### Chemical crosslinking of purified NS1-2

NS1-2 samples were collected from the size exclusion column in citrate-phosphate buffer and prepared to a final concentration of 0.05 mg/ml. Each protein sample was cross-linked with GA (0.005%–0.01% for 10 min at 37°C). The crosslinking reactions were stopped by incubating with 100 mM Tris pH 8 and samples were mixed with 2× SDS-PAGE sample buffer, boiled and analysed by SDS-PAGE and western blot.

### Bacterial two-hybrid assay

The interaction between NS1-2 monomers was assayed using the BacterioMatch® II two-hybrid system (Stratagene). This assay measured the interaction between the NS1-2 protein fused to the RNA polymerase α-subunit in the target plasmid (pTRG) and the NS1-2 protein fused to the bacteriophage λcl protein in the bait plasmid (pBT). Full-length and truncated NS1-2 clones were generated in each plasmid and protein expression tested in the reporter *E. coli* strain as per the manufacturer's instructions. Western blot analysis using the rabbit NS1-2 antibody was used to verify the NS1-2 expression. Calcium competent BacterioMatch® reporter cells (100 µl) were co-transformed with 50 ng of each plasmid and processed and plated onto selective medium containing 3 mM 3-amino-1,2,4-trizole according to the manufacturer's instructions.

### Expression of NS1-2 in HEK293T cells

Full-length NS1-2 and truncated NS1-2 constructs were generated under the control of a CMV promoter in pCMVSport1. HEK293T cells (CRL-11268™, ATCC™) were prepared in a 6-well tissue culture dish to reach ∼90% coverage prior to transfection. Transfection was achieved using Fugene® HD transfection reagent (Roche) as per manufacturer's instructions. Briefly, for each well, 2 µg of DNA was added to DMEM + GlutaMax™-1 (Invitrogen, Carlsbad, CA, USA) to give a total volume of 100 µl. Fugene® HD (6 µl) was added to the DNA/DMEM mix and incubated for 15 minutes at room temperature. This was then added to the prepared HEK293T cells and incubated at 37°C with 5% CO_2_ for 24 hours.

### Chemical crosslinking of transfected HEK293T cells

At 24 hours post-transfection, the medium from the HEK293T cells was removed and each well washed with 2 ml of DPBS (Dulbecco's phosphate buffered saline) (Oxoid Ltd., Hampshire, UK). Trypsinisation was achieved with 0.5 ml of 2.5 mg/ml trypsin in DPBS containing 0.4 mM EDTA, before neutralising with 0.5 ml DPBS. Cells were pelleted at 500×g for 5 minutes, washed twice with PBS(DSS) (20 mM sodium phosphate pH 8, 150 mM NaCl) and resuspended in PBS(DSS) at ∼25×10^6^ cell/ml. Crosslinking was obtained by incubating cells with 5 mM DSS for 30 minutes at room temperature, before stopping the reaction with 15 mM Tris pH 7.5. Samples were mixed with 2× SDS-PAGE sample buffer, boiled and analysed by SDS-PAGE and western blot.

### Sucrose gradient assay

5 ml linear sucrose gradients (5–20%) were prepared in SW55 tubes (Beckman-Coulter, Brea, CA, USA) using a gradient mixer and cooled to 4°C prior to loading the protein samples and standards. Three protein standards were prepared to 5 mg/ml in 20 mM citrate-phosphate pH 6.1, 150 mM NaCl – alcohol dehydrogenase, albumin and lysozyme. NS1-2trunc samples (monomer and dimer) were collected from the size exclusion column and prepared to 1 mg/ml. Solutions were then prepared that contained 200 µg of NS1-2 and 100 µg of each standard and centrifuged for 15 minutes, 16,000×g, 4°C prior to applying to the sucrose gradient. Gradients were centrifuged for 16 hours at 130,000×g and 4°C. Fractions of 400 µl were harvested from the bottom of the gradients and visualised by SDS-PAGE. The fraction number (#) most closely corresponding to the centre of the protein spread for each standard and NS1-2trunc sample was determined. Plotting the sedimentation coefficient (S) versus fraction number for each standard generated the linear equation of 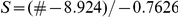 (r^2^ = 0.9997). The S values for the NS1-2trunc monomer and dimer were determined from this equation.

### Hydrodynamic characterisation

Calibration of the size exclusion column allowed us to determine the Stokes Radius (R_s_) of the NS1-2 protein using the following equation; 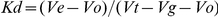 [48]. The values specific for the Superose12 column were V_o_ (void volume) = 7.59 ml, V_t_ (total volume of column set up) = 24.65 ml, V_g_ (gel matrix volume) = 5.15 ml. V_e_ (elution volumes) were determined for each standard and NS1-2 protein sample. The K_d_ value was calculated for each standard and K_d_^1/3^ was plotted versus R_s_, resulting in the linear equation of 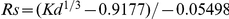. R_s_ values for each of the expressed regions of the NS1-2 protein were determined from this standard curve.

Uversky [49] has shown that different protein conformations can be calculated according to the equations shown below. The R_s_ values for each of the expressed regions of the NS1-2 protein were predicted for each of these conformation options: Native conformation, 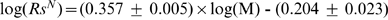; Molten globule, 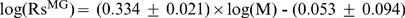; Premolten globule, 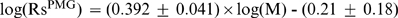; Unfolded in Urea, 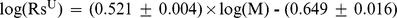.

The simplified Svedberg equation of 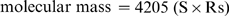 (Equation 7.1b [47]) was used to determine the experimental measure of the molecular mass of the NS1-2trunc monomer and dimer. The determination of the S_max_/S ratio for a protein indicates the shape of the protein in solution. Globular proteins have ratio values between 1.2 and 1.3, moderately elongated proteins have values between 1.5 and 1.9 and highly elongated proteins have values between 2.0 and 3.0 [47]. The S_max_ (maximum possible sedimentation coefficient) values for the NS1-2trunc monomer and dimer were calculated using the simplified equation of 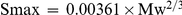 (equation 4.3b [47]). The S_max_/S ratios were then determined for each.

### Circular dichroism (CD)

CD spectra were recorded at 20°C on an Olis® (Bogart, GA, USA) CD module equipped with a Quantum Northwest temperature control system and the data was collected using the Olis® GlobalWorks™ software package. The proteins were prepared in 20 mM citrate-phosphate pH 6.1, 150 mM NaCl at a concentration of 0.2 mg/ml and 0.3 mg/ml for NS1-2trunc and NS1-2casp respectively. CD spectra were measured between 190 and 260 nm in a 1 mm cuvette and averaged from five scans. The contribution of buffer was subtracted from experimental spectra. The mean residue weight (MRW) was calculated by MRW  =  molecular weight [Da]/(number of residues – 1). The mean ellipticity values [θ] were calculated by [θ]  =  (millidegrees value × MRW)/(pathlength [mm] × concentration [mg/ml]). The experimental data in the 190–260 nm range were analysed using the DichroWeb online CD server (http://dichroweb.cryst.bbk.ac.uk) [53], which is supported by a grant from the Biotechnology and Biological Sciences Research Council. The CDSSTR [50], SELCON3 [52] and CONTIN [51] deconvolution methods were used to estimate the α-helical and β-sheet content using the reference dataset 7 (optimised for 190–240 nm).

### Dynamic light scattering analysis of NS1-2

Dynamic light scattering experiments were performed at 4°C in a Protein Solutions DynaPro™ (Wyatt Technology, Santa Barbara, CA, USA). Protein samples were prepared at 1 mg/ml in 20 mM citrate-phosphate pH 6.1, 150 mM NaCl. The samples were clarified prior to analysis by centrifuging at 16,000×g for 10 minutes at 4°C. The hydrodynamic radii were determined using the Dynamics V6 software (Wyatt).
